# UV-irradiated rotifers for the maintenance of gnotobiotic zebrafish larvae

**DOI:** 10.1128/msphere.00698-24

**Published:** 2025-02-24

**Authors:** Susana Márquez Rosales, Peter I. Bouchard, Emily M. Olmstead, Raghuveer Parthasarathy

**Affiliations:** 1 Department of Physics, Institute of Molecular Biology, and Materials Science Institute, University of Oregon, Eugene, Oregon, USA; The University of Iowa, Iowa City, Iowa, USA

**Keywords:** zebrafish, rotifers, gnotobiology

## Abstract

Host-associated microbial communities profoundly impact the health of humans and other animals. Zebrafish have proven to be a useful model for uncovering mechanisms of host-microbe interactions, but the difficulty of maintaining germ-free or gnotobiotic zebrafish beyond 1 week post-fertilization has limited their utility. To address this, we have developed a simple protocol using UV irradiation of rotifers, a common and nutrient-rich prey species for larval zebrafish, to reduce the bacterial load associated with the rotifers by several orders of magnitude while maintaining their motility and viability. We find that though feeding with UV-treated rotifers does not preserve the sterility of germ-free fish, it enables the maintenance of pre-existing bacterial communities. Normal feeding, in striking contrast, leads to the near-total depletion of these prior populations. We measure the abundance of single- and three-species consortia of zebrafish-commensal bacteria inoculated into initially germ-free larvae in a series of experiments extending to 8 days of feeding, or 13 days post-fertilization. We find, in fish-fed UV-treated rotifers, the persistence of bacterial populations on timescales of days, together with strong species-specific variation. In addition, re-inoculation of differently labeled strains of the same zebrafish-commensal species alongside feeding leads to colonization by the new bacteria without displacement of earlier microbes. Our method will facilitate the use of gnotobiotic zebrafish for investigations of phenomena that emerge later in animal development and for studies that probe microbiome composition fluctuations and stability over extended timescales.

**IMPORTANCE:**

All animals, including humans, are host to vast microbial communities that contribute to health and disease through mechanisms that remain largely mysterious. These microbiomes are challenging to study, spurring the use of various model organisms, including zebrafish. Zebrafish, however, are difficult to raise beyond 1 week post-fertilization under gnotobiotic conditions, in other words, germ free or with known microbial constituents, a consequence of normally feeding on live prey that brings their own, generally unknown, microbes. Therefore, we developed a simple protocol in which UV irradiation of rotifers, a widely used small-animal food for larval zebrafish, facilitates the maintenance of gnotobiotic larvae. We show that pre-existing bacterial communities in larvae are minimally affected by feeding on UV-treated rotifers, in strong contrast to feeding on untreated rotifers. We demonstrate that this feeding method allows investigations of zebrafish-associated bacterial community stability over several days, allowing investigation of previously intractable questions about microbiome stability.

## INTRODUCTION

The human body sustains nearly 100 trillion microorganisms, most of which form dense communities in the intestinal tract, communicating with host organs and influencing the course of health and disease (1). Manipulating and controlling microbial variables to uncover mechanisms of host-microbe interactions is challenging in humans, which has long motivated the use of animal models and the development of strategies to raise them germ free, i.e., devoid of microorganisms, or under gnotobiotic conditions, i.e., with known microbial strains or communities. Reports of germ-free guinea pigs date from 1896 (2), and since then, researchers have successfully derived various germ-free animals, including mammals, birds (3), amphibians (4), and fish. In 1942, Baker et al. reported the first germ-free fish (platyfish, *Xiphophorus maculatus*) (5), which was followed by other aquatic species including zebrafish (*Danio rerio*) (6, 7), a species whose attributes such as rapid development, genetic tractability, and transparency at young ages have made it a widely used animal model (8–10). Zebrafish-based studies of host-microbe interactions have illuminated the varied roles of commensal microbes in early development (9) including effects on epithelium maturation, secretory cell proliferation, enzymatic activity (6, 11–13), immune cell numbers (14–16), the abundance of insulin-producing β cells (17, 18), and neural anatomy influencing behavior (19). Cell-resolved live imaging of gut microbes in larval zebrafish (20), so far impossible in other vertebrates, together with a library of fluorescently labeled zebrafish-native bacterial species (21), has revealed diverse aggregation states among gut microbes (22–24), influences of bacterial motility on host immune response (25), effects of antibiotics on bacterial motility and persistence (26), and non-pairwise interactions among gut bacterial species (27).

One major aspect of microbiome research to which zebrafish has contributed far less as a model is the investigation of long timescales, necessary for studying bacterial community stability and changes over developmental periods extending to the whole lifespan. This is important because studies in humans show significant variations of timescales bacterial persistence throughout a host’s life, ranging from mere days or weeks, particularly during early developmental stages (28), to extended periods stretching up to months or even years in adults (29–31). Perturbations like changes in diet or antibiotic introduction can also alter the microbiota on timescales of days or weeks, though a full return to the original state can take years (32–35). For all these temporal changes, the underlying mechanisms are poorly understood (36). Zebrafish could help illuminate these issues for the reasons noted above, but they have suffered from a key limitation: the difficulty of maintaining gnotobiotic animals beyond approximately 7 days post-fertilization (dpf), at which point, having exhausted their yolk, they require food. While it is possible to provide sterilized, powdered chow (6), zebrafish larvae raised on dry food show diminished growth and development compared to larvae fed live food (37). Larval zebrafish are primarily visual hunters, and the motion of prey is crucial to stimulating capture and food intake (38, 39). Several research groups have therefore pursued the derivation of germ-free prey species. These include *Artemia* (brine shrimp), which researchers since the 1950s have made germ-free through egg sterilization (40–43). Recently, Jia et al*. (*44) showed that a different fish species (medaka, *Oryzias melastigma*) could be raised germ free to adulthood solely on *Artemia*. Zebrafish is capable of eating *Artemia* by 9 or 10 dpf, a few days after yolk depletion, calling for another, intermediate, prey species. For earlier feeding of zebrafish, groups have reported antibiotic treatment of the unconventional prey species *Tetrahymena thermophila* (41, 42). While successful for producing gnotobiotic fish, such methods involving these prey species, especially *Artemia*, are highly labor intensive (7) and are not widely used. Moreover, antibiotics, even at low concentrations, can have large impacts on the gut microbiome (26, 35) and can alter animal metabolism and growth (45), further motivating our efforts to investigate alternative methods. We consider rotifers (*Brachionus plicatilis*), an easily grown and widely used live food for zebrafish larvae (46, 47), and UV irradiation. Rotifers have been the target of axenic method development for over half a century at least (48), for example through sterilization of amictic eggs (49).

Though the aim of such efforts has usually been to facilitate studies of rotifers themselves rather than their use as prey, we note that because of their role as filter feeders, rotifers are capable of harboring a wide variety of environmental bacteria, including fish pathogens (50).

Regardless of the motivation for sterilization, UV sterilization of rotifers has rarely been investigated. In 1999, Munro et al. reported an UV irradiation protocol that reduced the bacterial load of rotifers by 99% and showed that these rotifers could be successfully fed to turbot (51). However, no investigations have been made the gnotobiotic status of fish fed with UV-irradiated food. Therefore, we aimed to establish a simple method based on UV-exposed rotifers that can maintain gnotobiotic zebrafish for extended durations. We developed and compared two separate procedures to minimize native bacterial populations in rotifers: antibiotic treatment and a simple UV irradiation protocol. We assessed the effect of consumption of treated rotifers on pre-existing bacterial populations in larval zebrafish. While both prey treatments reduced the rotifers’ bacterial load, consumption of antibiotic-treated rotifers led to dramatic drops in commensal bacterial abundance. In contrast, consumption of UV-treated rotifers left larval bacterial populations largely intact. Then, we examined bacterial populations in fed larvae to 13 dpf, demonstrating greater stability in fish fed with UV-treated rotifers than in untreated rotifers. The methods described here will facilitate studies of gut microbiome dynamics and host-microbe interactions in gnotobiotic animals.

## RESULTS

We aimed to establish an easily implemented method for feeding rotifers to zebrafish larvae while maintaining gnotobiotic conditions. The key step is to reduce as much as possible the bacteria that naturally accompany the rotifers while preserving rotifer survival and motility. We investigated two methods: one using UV light exposure and the other using antibiotics.

**Fig 1:**
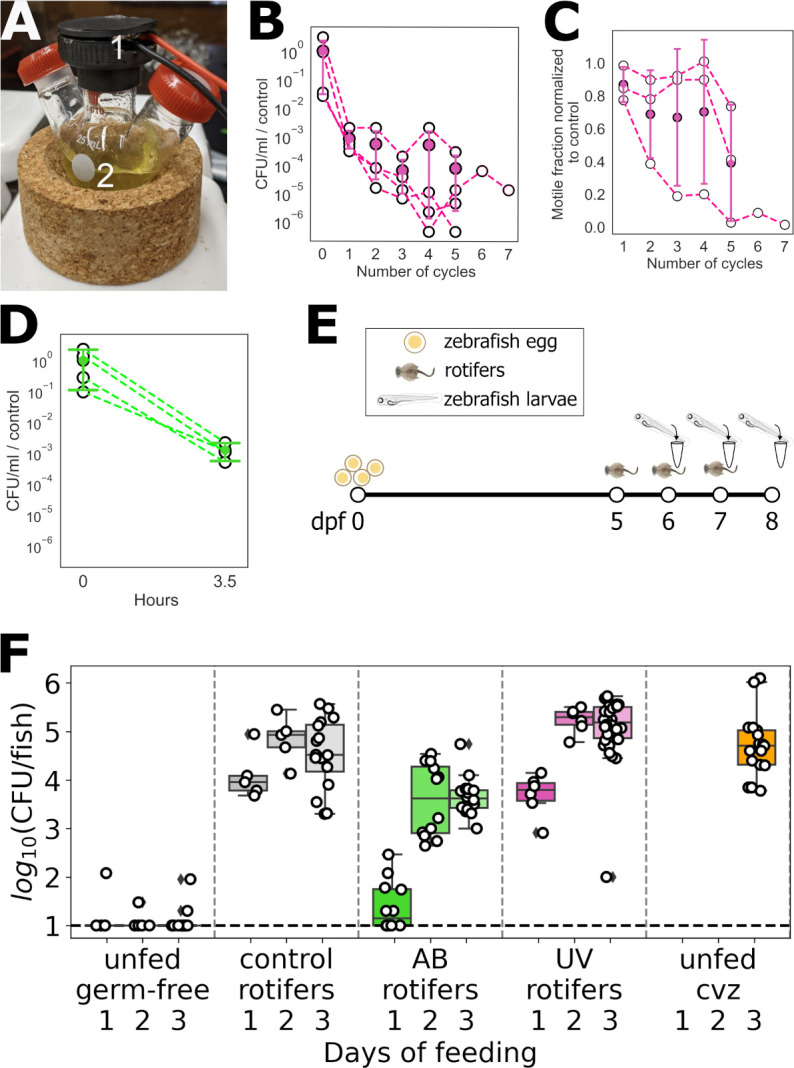
(A) Experimental setup for UV irradiation of rotifers, including (1) a UV-C LED mounted in a cap of a round bottom flask and (2) rotifers in a 4 ppt NaCl solution. (B) Bacterial concentration in the rotifer suspension as a function of the number of UV exposure cycles, each 30 minutes on/30 minutes off. (C) Fraction of motile rotifers as a function of the number of UV exposure cycles. In panels B and C, open symbols represent different replicates, and solid symbols and error bars indicate the mean and SD. (D) Bacterial concentration in the rotifer suspension before and after incubation with antibiotics for 3.5 hours. (E) Schematic diagram of the protocol for assessing bacterial load in initially germ-free zebrafish subject to different feeding methods. (F) Bacterial abundance per larval zebrafish after 1, 2, and 3 days of feeding with rotifers under different treatments, or unfed at the same days. Each symbol indicates a measurement from an individual fish; boxes indicate the median (line within the box), first and third quartiles (top and bottom of the box), and 95% confidence intervals (bars). The dashed line indicates the approximate limit of detection, 10 bacteria per fish.

The UV irradiation method uses a 270–280 nm UV-C LED attached to the cap of a flask containing a rotifer suspension and a stir bar to ensure mixing (Fig. 1A; see Materials and Methods). To find an exposure protocol that leads to a significant reduction in bacterial numbers while maintaining a viable rotifer population, we assessed cycles of UV light consisting of alternating 30-minute exposure and rest periods. By three exposure cycles, plating the rotifer suspension showed a drop in bacterial abundance of nearly four orders of magnitude (Fig. 1B).

UV irradiation causes rotifers to become slow or non-motile. By five UV cycles, the fraction of motile organisms decreased to less than 50%, with the non-motile animals lacking apparent internal organ activity and therefore likely being dead, and the average speed of motile rotifers was less than half that of unexposed rotifers (Fig. 1C; Fig. S1; Movies S1 and S2; see Materials and Methods). Therefore, we chose four UV exposure cycles to reduce the rotifers’ bacterial load while keeping the majority of animals motile (Fig. 1C) and therefore likely to stimulate the prey capture response of hunting zebrafish larvae.

While a large variety of antibiotics exist, we focused for simplicity on the same cocktail of antibiotics typically used for germ-free zebrafish derivation (7) (see Materials and Methods). After 3.5 hours of incubation, the antibiotics reduced the bacterial load of rotifer suspensions by three orders of magnitude (Fig. 1D), similar to the results from UV irradiation. Following antibiotic treatment, we observed no impact on rotifer swimming speed or the proportion of motile rotifers.

We evaluated the efficacy of the UV and antibiotic treatments for maintaining germ-free fish after feeding. Beginning with germ-free larvae, we administered feeding regimes spanning 1, 2, and 3 days starting at 5 dpf (Fig. 1E), followed by bacterial abundance quantification via larval homogenization and plating (see Materials and Methods). Notably, we measured bacterial load from entire larvae, which will be dominated by gut microbes but can include skin-resident microbes. Figure 1F presents the results detailing bacterial loads from larvae fed with UV-treated rotifers, antibiotics-treated rotifers (AB), and control (untreated) rotifers, alongside unfed germ-free fish and unfed conventionalized larvae, the latter initially germ-free and then raised in standard, non-sterile aquaculture water. As expected, unfed conventionalized larvae and larvae fed control rotifers showed 10^4^–10^5^ bacteria (colony-forming units [CFU]) per fish, while unfed germ-free fish typically had undetectable numbers of bacteria (Fig. 1F). Fish fed with AB-treated rotifers showed low bacterial abundance after 1 day of feeding (10–100 CFU), though bacterial numbers increased to several thousand by 2 or 3 days of feeding. Fish fed with UV-treated rotifers lost their germ-free status after just 1 day of feeding, with bacterial abundance around 10^4^ indicating that the small amount of bacteria remaining in the rotifers after UV treatment are sufficient to colonize the larvae, compromising the gnotobiotic conditions. We also measured larval length after 2 or 3 days of feeding as an indicator of fish health. Larvae in all fed groups were, on average, longer than unfed larvae (Fig. S2). The length of fish fed with UV-treated rotifers showed no significant difference compared to those fed with control rotifers, implying that the decrease in motility and speed of rotifers due to UV treatment did not negatively affect their capture by hunting fish.

**Fig 2:**
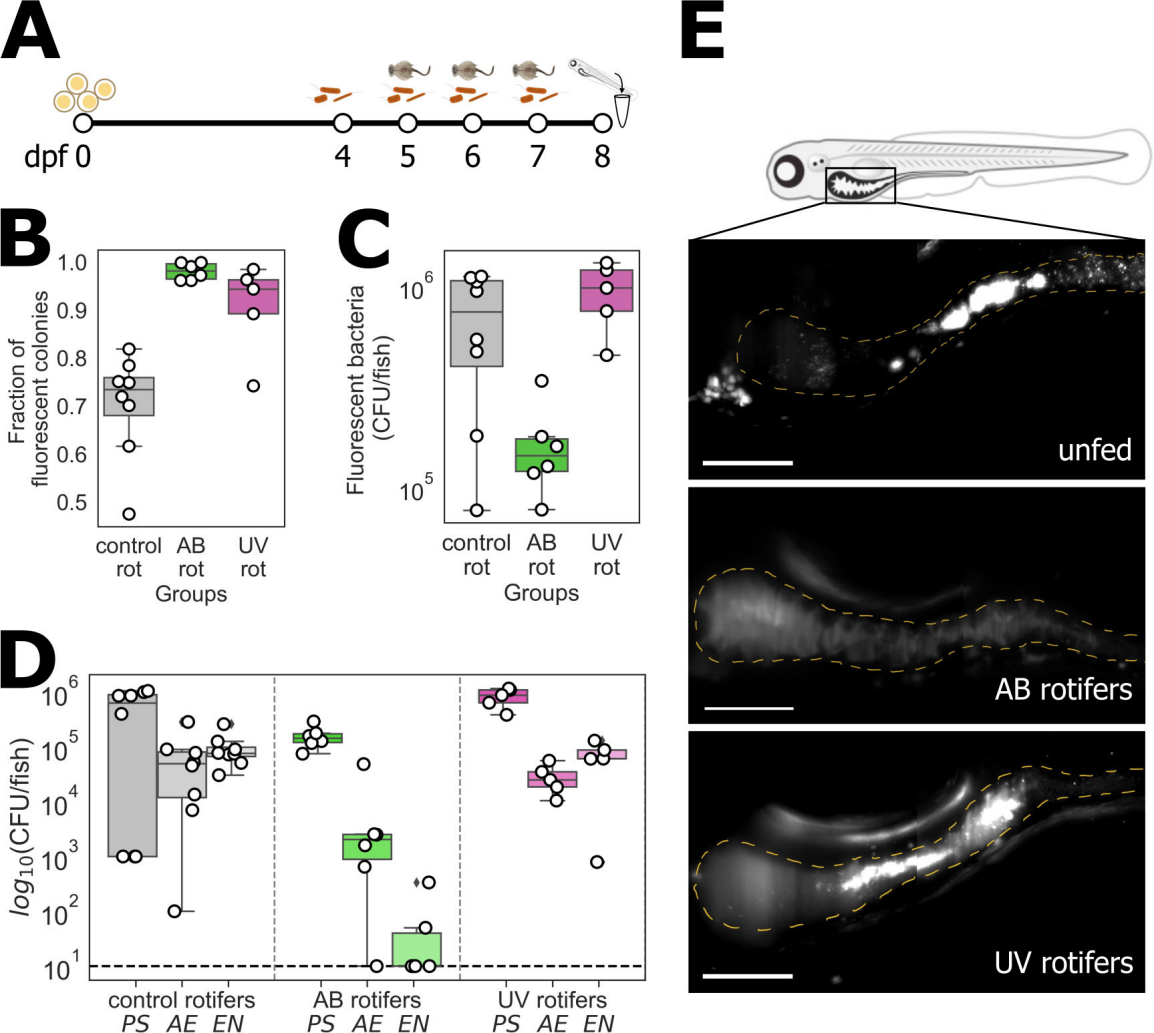
(A) Schematic diagram of the protocol to evaluate the permanence of gnotobiotic larvae after 3 days of feeding, in which initially germ-free zebrafish is inoculated with three fluorescently labeled bacterial species. (B) Fraction of fluorescent colonies from plating whole larvae, indicating the fraction of intentionally inoculated bacteria, after 3 days of feeding and re-inoculation. (C) Total abundance of fluorescent bacteria per larva after 3 days of feeding and re-inoculation. (D) Abundance of each intentionally inoculated strain. PS: *Pseudomonas mendocina* ZWU0006, AE: *Aeromona veronii* ZOR0001, EN: *Enterobacter cloacae* ZOR0014. In panels B–D, each symbol indicates a measurement from an individual fish; boxes indicate the median and quartiles. (E) Maximum intensity projections of representative 3D image stacks from light sheet fluorescence microscopy of the guts of live larval fish, taken after 1 day of feeding with antibiotic- or UV-treated rotifers, or on the equivalent day for unfed fish. Each image is assembled from four or five scans of sub-regions that together span the whole gut, computationally registered after acquisition as described in Materials and Methods. Dashed lines trace approximate intestinal boundaries. Bar = 200 µm.

Though the methods being examined cannot maintain zebrafish germ free, they nonetheless may be capable of keeping larvae under gnotobiotic conditions, sustaining intentionally inoculated bacterial communities with minimal perturbation. We hypothesized that symbiotic strains may out-compete the bacteria that accompany rotifers. To assess this, as indicated schematically in Fig. 2A, we started by establishing a bacterial community at 4 dpf by inoculating germ-free larval fish with previously studied zebrafish-native strains expressing fluorescent proteins (see Materials and Methods) (21, 27) *Pseudomonas mendocina* ZWU0006 (PS), *Aeromona veronii* ZOR0001 (AE), and *Enterobacter cloacae* ZOR0014 (EN). On each of the next 3 days (5–7 dpf), we fed the fish and also re-introduced the fluorescent strains to the aqueous medium. At 8 dpf, we homogenized and plated the whole fish as above (see Materials and Methods), noting the abundance of fluorescent colonies, representing the intentionally inoculated species, and non-fluorescent colonies, representing the species introduced by feeding.

We found that for fish fed with AB- and UV-treated rotifers, the abundance of fluorescent bacteria was over 90% of the total bacterial load (0.98 ± 0.016, mean ± SD, and 0.90 ± 0.087, respectively; Fig. 2B). In contrast, the abundance of fluorescent bacteria in fish fed with control rotifers was considerably smaller, 0.7 ± 0.1 of the total. Strikingly, the overall number of intentionally inoculated (fluorescent) bacteria was approximately an order of magnitude lower in fish fed with AB-treated rotifers than in fish fed with UV-treated or control rotifers (Fig. 2C). Plating on chromogenic agar, on which colonies of the different intentionally inoculated strains show different colors (27) (see Materials and Methods), showed that the depletion of bacterial numbers following feeding with AB-treated rotifers was strongly species dependent (Fig. 2D), with the EN population being particularly affected and completely absent in most larvae (Fig. 2D). The AE population was two orders of magnitude smaller compared to other fed groups, while PS was similar (Fig. 2D). To gain insights into intestinal abundance in particular, we imaged the whole intestines of live larvae using light sheet fluorescence microscopy (22, 24). We could not detect fluorescent bacteria in the gut of larvae fed with AB-treated rotifers, while fluorescent bacteria were clearly evident in larvae fed with UV-treated or control rotifers (Fig. 2E).

Having established a method using UV-irradiated rotifers to feed zebrafish larvae, we investigated inoculated *Enterobacter cloacae* (EN) populations over several days, noting that of the three species examined this strain was the most sensitive to food-induced perturbation (Fig. 2D). To distinguish bacteria descended from those inoculated on the first day, colonizing germ-free fish, from bacteria introduced on subsequent days, we used differently tagged EN strains (21), expressing green fluorescent protein (GFP) and dTomato, respectively. As above, non-fluorescent bacteria represented unintended colonizers from rotifers or other sources. Fish were fed from 5 to 7 dpf or from 7 to 9 dpf. The first set allowed us to include unfed fish as a control group. The second set tested bacterial dynamics into the regime that requires feeding. For both sets, we included fish fed with control (untreated) rotifers to determine the efficacy of the UV treatment.

**Fig 3:**
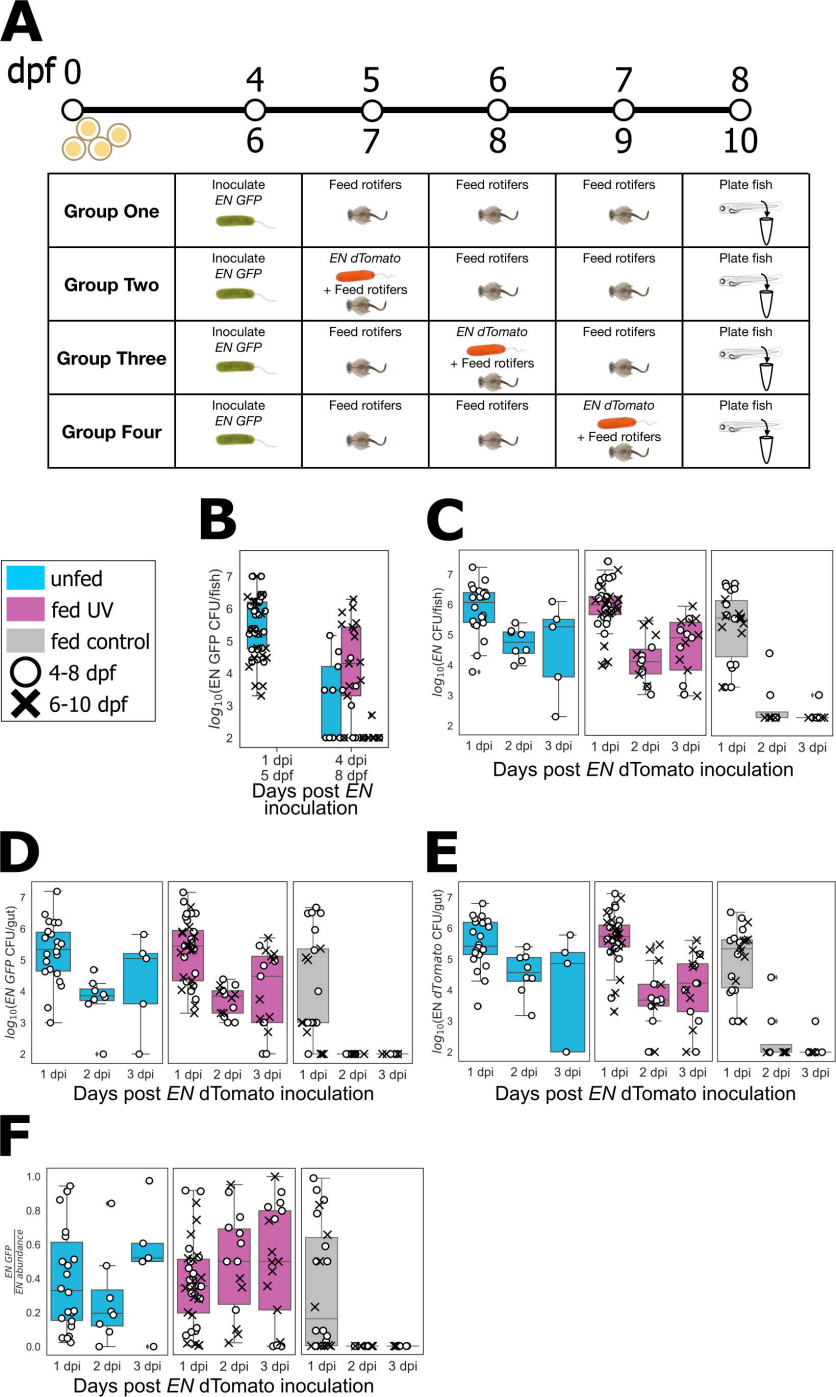
(A) Schematic diagram of the protocol to evaluate *Enterobacter* stability after feeding and re-inoculation. As described in the main text, initially germ-free fish were inoculated with GFP-labeled EN and fed UV-treated or control rotifers or left unfed for 3 days, starting at 4 or 6 dpf. Groups 2–4 were re-inoculated with dTomato-labeled EN on different days. (B–F) Final EN abundance in unfed larvae (cyan), larvae fed with UV-treated rotifers (pink), and larvae fed with untreated rotifers (gray). Circles represent data from larvae fed from 5 to 7 dpf and crosses from 7 to 9 dpf. (B) Fluorescent EN-GFP abundance in group 1 larvae assessed 1 day post-inoculation (before the start of feeding) and 4 days post-inoculation (after 3 days of feeding). (C–E) From groups 2, 3, and 4, total fluorescent EN abundance (GFP and dTomato) (C), EN-GFP abundance (D), and EN-dTomato abundance (E) 1, 2, or 3 days post EN dTomato inoculation. (F) Fraction of EN-GFP relative to the total EN abundance 1, 2, or 3 days post EN-dTomato inoculation.

The study comprised four distinct fish groups for each set of days, as illustrated in Fig. 3A. All fish were inoculated on the initial day (4 or 6 dpf) with GFP-labeled EN, fed with UV-treated or control rotifers on each of the next 3 days unless in the unfed set, and then plated to determine bacterial load on various days including the final day (Fig. 3A). Groups 2, 3, and 4 were, in addition, inoculated with dTomato-labeled EN on the first, second, and third day of feeding, respectively (Fig. 3A; see Materials and Methods). Representative larvae were homogenized and plated as above to quantify bacterial load, with all fish assessed by the final day, at 8 or 10 dpf. Together, these groups provided insight into the persistence of EN introduced before feeding and of stability following re-inoculation.

We present the results in Fig. 3, indicating the feeding types by different colors and the sets of days by different symbols. Considering fish only inoculated on the first day, by EN-GFP, the initial bacterial load assessed from unfed fish at 1 day post-inoculation (dpi) was roughly 10^5^–10^6^ per fish, as expected (Fig. 3B). At 4 dpi, almost no EN was found in fish fed with untreated rotifers, in contrast to roughly 10^3^–10^5^ in fish fed with UV-treated rotifers. Interestingly, unfed fish at 8 dpf showed fewer EN (Fig. 3B), suggesting that the depletion of yolk has consequences for resident bacteria. We next considered the total EN population in groups 2, 3, and 4, as a function of days following inoculation of the second (EN-dTomato) bacterial strain (Fig. 3C). Again, almost no EN was found in fish fed with untreated rotifers at 2 and 3 dpi, while unfed fish and fish fed with UV-treated rotifers showed sizable bacterial numbers (10^4^–10^5^ per fish; Fig. 3C). Considering separately the EN-GFP population, descended from the initial colonizers, and the EN-dTomato population, descended from later entrants, we found similar orders of magnitude for the abundance of each group in the unfed fish and fish fed with UV-treated rotifers (Fig. 3D and E). The relative abundance of the two groups, quantified as the GFP-labeled fraction of the total EN abundance, showed considerable variation (Fig. 3F), indicating that neither the initial nor later colonizers consistently dominates the population.

**Fig 4:**
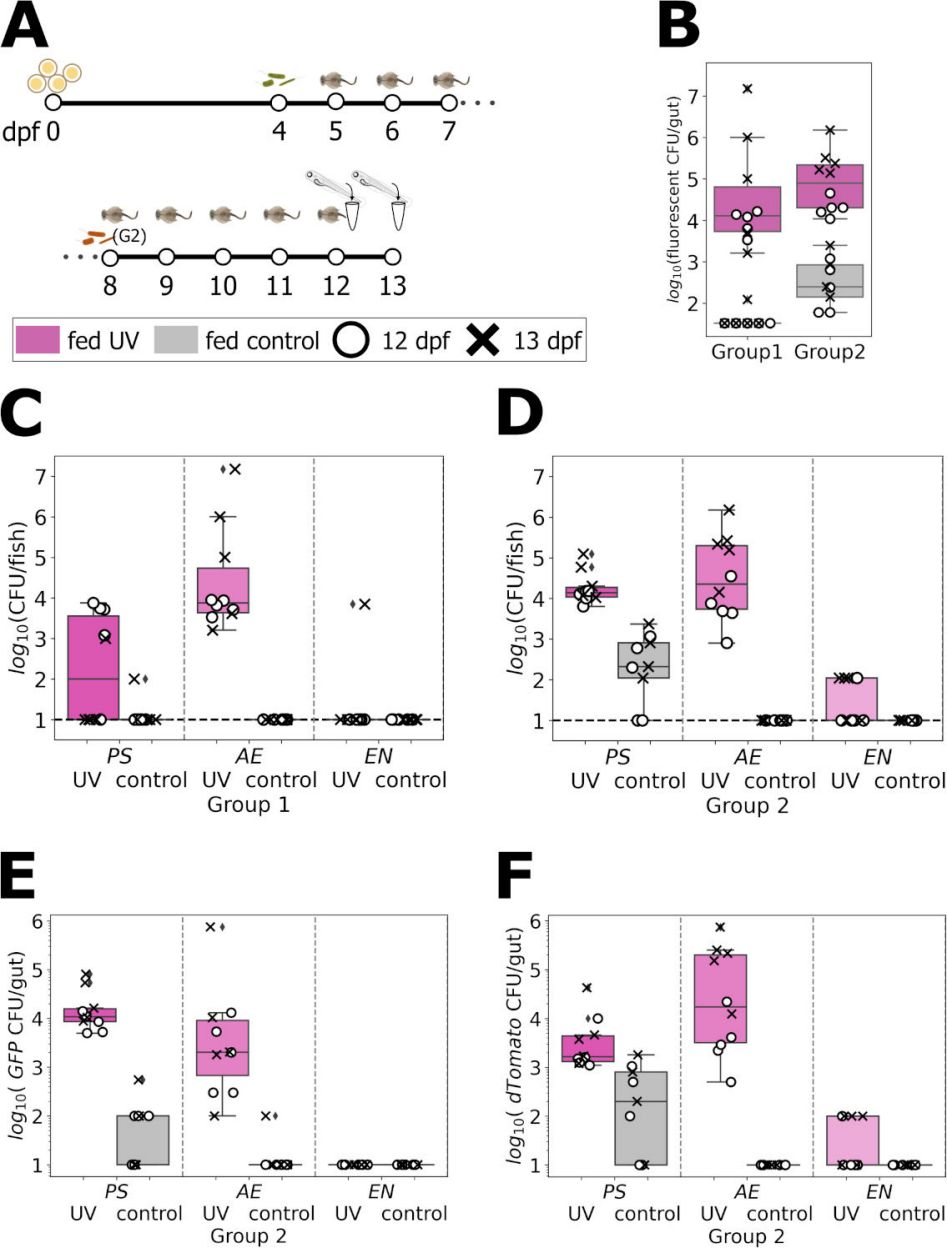
(A) Schematic diagram of the protocol to evaluate the stability of multiple bacterial species in larvae fed until 13 dpf. Initially germ-free larvae were inoculated with GFP-labeled PS, AE, and EN at 4 dpf and split into two groups. Zebrafish in group 2 was re-inoculated with dTomato-labeled PS, AE, and EN at 8 dpf. Both groups were fed from 5 to 11 or 12 dpf and homogenized and plated at 12 or 13 dpf. (B) Fluorescent bacterial abundance per larva after 7 or 8 days of feeding. Pink corresponds to larvae fed with UV treated rotifers and gray to larvae fed with untreated rotifers. (C and D) Bacterial abundance of each inoculated per strain for fish in groups 1 (C) and 2 (D). (E) Total abundance of GFP-labeled bacteria, descended from initial colonizers, of each strain in larvae from group 2. (F) Total abundance of dTomato-labeled bacteria, descended from later colonizers, in larvae from group 2.

Finally, we examined the stability of a multi-species bacterial consortium in zebrafish up to 13 dpf, several days longer than is possible without feeding. We again considered PS, AE, and EN (as in Fig. 2) and again considered differently inoculated groups. We inoculated all larvae with GFP-labeled PS, AE, and EN at 4 dpf and group 2 larvae additionally with dTomato-labeled PS, AE, and EN at 8 dpf, the fourth day of feeding (Fig. 4A; see Materials and Methods). Both groups were fed for 7 or 8 days after which individuals were homogenized and plated to assess bacterial load. Both groups included fish fed with UV-treated rotifers and control (untreated) rotifers.

Considering the total abundance of fluorescent (intentionally inoculated) bacteria at the experimental endpoint, fish in groups 1 and 2 that were fed UV-treated rotifers showed sizable populations, roughly 10^4^–10^5^ CFU/fish, while fish fed with control rotifers showed bacterial numbers that were on average two orders of magnitude lower (Fig. 4B). Quantifying each inoculated bacterial species separately showed considerable differences (Fig. 4C and D). AE was the most stable species, with abundances similar to those measured 1 day after bacterial inoculation in earlier experiments (Fig. 2D), and EN was the least stable species. For all species, abundances in control-rotifer-fed fish were low or undetectable (Fig. 4C and D). Considering separately, in group 2 fish, the GFP-labeled population, descended from the initial colonizers, and the dTomato-labeled population, descended from later entrants, we found similar orders-of-magnitude for the abundance of each in the fish fed with UV-treated rotifers (Fig. 4D and E). Intriguingly, PS and AE showed opposite behaviors for the abundance of dTomato- relative to GFP-labeled bacteria, with PS dominated by the descendants of initial colonizers and AE the later colonizers (Fig. 4E and F). Variation was large, however, prohibiting strong conclusions.

## DISCUSSION

To improve the utility of zebrafish as a model for studying animal-associated microbiomes, we have developed a method for feeding zebrafish larvae that allows live, motile food while minimizing the disruption of existing bacterial communities. Our method uses UV-C LEDs that are inexpensive, easy to deploy, and sufficient to reduce within a few hours the bacterial density that naturally accompanies rotifers by four orders of magnitude. Although maintaining truly germ-free zebrafish larvae was not achievable with this method of preparing prey (Fig. 1F), likely due to the ability of remaining microbes to proliferate in the sterile larval environment, we found that feeding with UV-irradiated rotifers allows zebrafish-native bacteria associated with the fish prior to feeding, or reintroduced together with feeding, to persist at high abundance (Fig. 2 and 3).

Importantly, the similarity between the abundances of intentionally inoculated bacteria in unfed fish and those fed with UV-treated rotifers, and the marked contrast with fish fed with untreated rotifers, validates the UV treatment protocol for studying zebrafish-associated bacterial stability. Normal feeding on untreated rotifers, in contrast, diminishes fish native bacteria by orders of magnitude.

Treatment of rotifers with an antibiotic mixture commonly used for deriving germ-free fish, though effective for reducing the bacterial concentration in the rotifer suspension (Fig. 1D) leads after ingestion to large and species-specific drops in fish-associated bacterial abundance (Fig. 2C and D). This is perhaps unsurprising, as the antibiotics are diluted only by a factor of 15 from the concentration used for deriving germ-free fish (see Materials and Methods). It may be possible, for example through filtration or centrifugation, to separate the rotifers from their antibiotic treated suspension prior to feeding, but this adds extra procedural steps, and moreover, it is possible that the antibiotics are preferentially concentrated in the rotifers. Furthermore, studies of humans (52) and zebrafish (26) have shown that even low doses of antibiotics can have large consequences on gut microbiomes, in part due to antibiotic-induced changes in bacterial morphology and motility (26), providing further cautions for antibiotic-based procedures for gnotobiotic prey. We believe, therefore, that our use of UV irradiation highlights a gnotobiotic strategy that is less likely to lead to strong perturbations of animal-associated microbiomes, and we focus on further characterization of this method.

Extending the timescale over which zebrafish can be maintained under gnotobiotic conditions opens up new research opportunities. For example, week-old gnotobiotic zebrafish larvae have enabled insights into connections between gut bacterial species and innate immune responses (53), but studying the adaptive immune system requires older fish, as the maturation of the adaptive immune system in zebrafish starts at 3 weeks post-fertilization with the appearance of CD4+/CD8+ lymphocytes (54). Given increasingly appreciated connections between host-associated microbes and autoimmune and chronic inflammatory disorders (55), older gnotobiotic zebrafish could serve as valuable experimental tools.

Most generally, improved gnotobiotic methods enable the extension of timescales of experimental investigation in zebrafish. Of particular interest are timescales of gut microbiome stability and instability, whose underlying determinants in humans and other animals remain mysterious (36). Given the presence of a bacterial strain, what is the likelihood of its persistence some time later, and is this altered by immigration of members of the same or different strains?

We have demonstrated that studying these questions is tractable, for example, examining a three-species consortium in larvae fed for up to 8 days, to 13 dpf. We find strong species dependence of stability, with a zebrafish-native *Enterobacter* nearly disappearing while other species persist (Fig. 4). Notably, this *Enterobacter* species also shows the greatest decline when fish are fed with antibiotic-treated rotifers (Fig. 2D). These observations are consistent with previous results from short-duration studies that show that *Enterobacter* is disfavored in competition with *Aeromonas* (23, 27) and is highly sensitive to weak antibiotics (26). *Enterobacter* is also highly aggregated (22), and we suspect that underlying all of these phenomena is a connection between aggregation and intestinal transport. With the ingestion of food, a frequent perturbation of obvious importance to humans and other animals, the physical space occupied by food particles and the mechanics of its digestion may induce species-specific displacement, particularly impacting more aggregated strains. Further experiments, including studies using live imaging (as in Fig. 2E), can directly probe and quantify these processes in a useful model animal.

## MATERIALS AND METHODS

### Animal care

All experiments with zebrafish were done in accordance with protocols approved by the University of Oregon Institutional Animal Care and Use Committee and by following standard protocols (56).

### Zebrafish gnotobiology

Wild-type ABC X TU zebrafish (*Danio rerio*) were derived germ-free as described previously (7) with slight modifications. In brief, fertilized eggs were collected and placed in sterile antibiotic embryo medium (EM) containing 100 µg/mL ampicillin, 250 ng/mL amphotericin B, 10 µg/mL gentamicin, 1 µg/mL tetracycline, and 1 µg/mL chloramphenicol for approximately 5 hours. The eggs were then washed in sterile EM containing 0.003% sodium hypochlorite and then in sterile EM containing 0.1% polyvinylpyrrolidone-iodine. Washed embryos were distributed into tissue culture flasks containing 50 mL of sterile embryo medium at a density of one embryo per. Flasks were inspected for sterility before being used in experiments.

### Bacterial strains

We used the following strains, each originally isolated from the zebrafish intestine: *Aeromonas* sp. strain ZOR0001, *Pseudomonas mendocina* (ZWU0006), and *Enterobacter* sp. strain ZOR0014 (EN). From each, engineered strains constitutively expressing GFP or dTomato were previously generated (21). Stocks of bacteria were maintained in 25% glycerol at ≤80°C.

### Inoculation of larval zebrafish

One day prior to fish inoculation, bacteria from frozen glycerol stocks were grown overnight in lysogeny broth (LB medium; 10 g/L NaCl, 5 g/L yeast extract, 12 g/L tryptone, and 1 g/L glucose) at 30°C with shaking. For inoculation of germ-free fish with a single species (*Enterobacter* sp. strain ZOR0014), 1 mL of overnight culture was washed once by centrifuging for 2 minutes at 7,000 × *g*, removing the supernatant, and adding 1 mL of fresh sterile embryo medium. Then 100 µL of the bacterial mix was added to a 50 mL tissue culture flask containing approximately 50 germ-free 4 dpf zebrafish larvae, giving a bacterial concentration of approximately 10^6^ CFU/mL. For experiments involving multiple bacterial species, we combined 1 mL of each overnight culture, diluting each as needed in lysogeny broth to achieve similar optical densities (OD600 ≈ 5.0) and therefore similar concentrations. The bacterial mixture was further prepared and added to the flasks containing larvae as above, at a bacterial concentration of approximately 10^6^ CFU/mL. For re-introduction of bacteria to previously colonized fish, we prepared a 1,000-fold dilution of the *Enterobacter* overnight culture in LB for single-species experiments and a 100-fold dilution of the bacterial mixture for multi-species experiments. Then, 100 µL of the bacterial suspension was combined with 900 µL of the rotifer suspension and added to the flasks containing zebrafish larvae as above, giving bacterial concentrations of approximately 10^4^ CFU/mL and 10^5^ CFU/mL (about 10^4^ CFU/mL per each species) for the single- and multi-species experiments, respectively.

### Rotifers

Rotifers (*Brachionis plicatilis*) were provided by the University of Oregon Zebrafish Facility. Rotifer cultures were raised in the facility in 5 gallon containers, fed with the “Rotigrow Plus” algae mixture (Reed Mariculture), and maintained at 4 ppt salinity in “Instant Ocean” commercial sea salt mix (Instant Ocean). Rotifers at a density of roughly 2,000/mL were obtained as needed from the facility and used the same day.

### UV irradiation of rotifers

UV light, 270–280 nm, was provided by LEDs (E275-3-S UVC LED Chip on Board, International Light Technologies, $16.85 each; UVC LED Driver ILT-PWRTYLED.3W, International Light Technologies, $21.91). An LED was glued to a 3D-printed cap designed to fit a three-neck round bottom flask (Synthware Round Bottom Flasks, Three Neck, Threaded, 25 mL; Fisher Scientific Catalog No.31-502-121); CAD files for the cap are provided in the Thingiverse repository, item no. 6737329 https://www.thingiverse.com/thing:6737329). The remaining necks of the flask, which allow easy extraction of the rotifer suspension, were kept loosely capped to allow oxygenation. For rotifer treatment, rotifers were diluted 5× in 4 ppt NaCl, reducing the opacity of the suspension, to a concentration of roughly 400/mL, and 15 mL of this suspension was placed in each round bottom flask.

The maximum depth of the suspension was 2.2 cm. A stir bar, 2 mm, was used for continuous stirring. The UV intensity was measured to be 0.48 mW/cm^2^ at the location of the water surface, 5.4 cm away from the LED.

### Antibiotic treatment of rotifers

We use the same antibiotic solution and concentrations noted in “Zebrafish gnotobiology” above, treating 1 mL of rotifer suspension for 3.5 hours.

### Rotifer motility assessment

Rotifer motility was assessed by tracking individual rotifers in brightfield movies captured at 10 frames per second with a scale of 6.5 µm/px. Using custom software, images were inverted, and regions of interest containing rotifers were identified as local intensity maxima. Rotifers were localized in each frame using a symmetry-based algorithm (57) and connected across frames using nearest-neighbor linkage. A rotifer was considered motile if the mean speed of its trajectory exceeded 50 µm/s, which clearly distinguished moving and stationary rotifers.

### Feeding

Before feeding, larvae were transferred to a new tissue culture flask with fresh sterile embryo medium. To 15 mL of the flask medium, we added 1 mL of rotifer suspension, corresponding to roughly 400 rotifers, or roughly 30 per fish. Fish were allowed to feed *ad libitum*.

### Light sheet fluorescence microscopy

Imaging was performed using a home-built light sheet fluorescence microscope based on the design of Keller et al. (58). The protocols to mount the fish and the details of the microscope can be found in references 59–61 and 25. In brief, we anesthetize larvae with MS-222 (tricaine, 20 µL/mL) and mount the fish in glass capillaries containing 0.7% agarose gel suspended vertically, head up, in a custom imaging chamber containing EM and MS-222. Lasers with excitation wavelengths of 488 and 568 nm were used to excite green fluorescent protein and dTomato labeled bacteria, respectively. To image the entire extent of the intestine covering the gut (approximately 1,200 × 300 × 150 µm) we move the specimen in *z*-steps of 1 µm and sequentially image four or five sub-regions computationally registering the images after acquisition. Registration is performed using custom software, publicly available in a GitHub repository (https://github.com/pamitabh/batch-processing/tree/main/user_friendly_downsampling_mip_stitch_batchprocess_code; function run2), which uses stage positions saved during image acquisition to align and stitch image stacks with the correct offsets.

### Plating and bacterial abundance measurement

To quantify bacterial abundance in the fish, we euthanized the fish via hypothermic shock and transfer single larva into 1.5 mL Eppendorf tubes containing 1 mL of sterile embryo medium and approximately 0.1 mL of 0.5 mm zirconium oxide pellets. Whole fish were homogenized with a bullet blender (Next Advance) for 3 minutes at speed 8. Dilutions of 10^−1^ and 10^−2^ were prepared, and 100 µL of each were spread on LB agar plates. CFUs on the plates were counted to quantify bacterial abundances. For experiments with zebrafish inoculated with more than two species, homogenized larvae were plated on Universal HiChrome agar (Sigma Aldrich) to distinguish species based on a colorimetric indicator. All plating-derived abundance data are provided as Supplemental Material.

## SUPPLEMENTAL MATERIAL

Supplemental Figures and Movie Captions msphere.00698-24-s0001.pdf Figures S1 and S2 and captions for Movies S1 and S2.

Supplemental Material msphere.00698-24-s0002.csv All plotted data points.

Movie S1 msphere.00698-24-s0003.avi Rotifers without UV irradiation.

Movie S2 msphere.00698-24-s0004.avi Rotifers following four cycles of UV irradiation.

ASM does not own the copyrights to Supplemental Material that may be linked to, or accessed through, an article. The authors have granted ASM a non-exclusive, world-wide license to publish the Supplemental Material files. Please contact the corresponding author directly for reuse.

## References

[1] Haller D. 2018. The gut microbiome in health and disease. Springer.

[2] Nuttall GHF, Thierfelder. H. 1897. Thierisches leben ohne bakterien im verdauungskanal. Hoppe-Seyler´s Zeitschrift für physiologische Chemie 23:231–235. doi:10.1515/bchm2.1897.23.3.231

[3] Reyniers JA, Trexler PC, Ervin RF, Wagner M, Luckey TD, Gordon HA. 1949. A complete life-cycle in the “germ-free” bantam chicken. Nature 163:67–68. doi:10.1038/163067a0 18106154

[4] Gordon HA. 1960. The germ-free animal. Its use in the study of “physiologic” effects of the normal microbial flora on the animal host. Am J Dig Dis 5:841–867. doi:10.1007/BF02232187 13707167

[5] Baker JA, Ferguson MS, TenBroeck C. 1942. Growth of platyfish (Platypoecilus maculatus) free from bacteria and other micro’organisms. Exp Biol Med (Maywood) 51:116–119. doi:10.3181/00379727-51-13854

[6] Rawls JF, Samuel BS, Gordon JI. 2004. Gnotobiotic zebrafish reveal evolutionarily conserved responses to the gut microbiota. Proc Natl Acad Sci USA 101:4596–4601. doi:10.1073/pnas.0400706101 15070763 PMC384792

[7] Melancon E, Gomez De La Torre Canny S, Sichel S, Kelly M, Wiles TJ, Rawls JF, Eisen JS, Guillemin K. 2017. Best practices for germ-free derivation and gnotobiotic zebrafish husbandry. Methods Cell Biol 138:61–100. doi:10.1016/bs.mcb.2016.11.005 28129860 PMC5568843

[8] Grunwald DJ, Eisen JS. 2002. Headwaters of the zebrafish — emergence of a new model vertebrate. Nat Rev Genet 3:717–724. doi:10.1038/nrg892 12209146

[9] Stagaman K, Sharpton TJ, Guillemin K. 2020. Zebrafish microbiome studies make waves. Lab Anim (NY) 49:201–207. doi:10.1038/s41684-020-0573-6 32541907 PMC7755162

[10] Milligan-McClellan K, Charette JR, Phennicie RT, Stephens WZ, Rawls JF, Guillemin K, Kim CH. 2011. Study of host–microbe interactions in zebrafish. Methods Cell Biol 105:87–116. doi:10.1016/B978-0-12-381320-6.00004-7 21951527 PMC4700925

[11] Bates JM, Mittge E, Kuhlman J, Baden KN, Cheesman SE, Guillemin K. 2006. Distinct signals from the microbiota promote different aspects of zebrafish gut differentiation. Dev Biol 297:374–386. doi:10.1016/j.ydbio.2006.05.006 16781702

[12] Troll JV, Hamilton MK, Abel ML, Ganz J, Bates JM, Stephens WZ, Melancon E, van der Vaart M, Meijer AH, Distel M, Eisen JS, Guillemin K. 2018. Microbiota promote secretory cell determination in the intestinal epithelium by modulating host notch signaling. Development 145:dev155317. doi:10.1242/dev.155317 29475973 PMC5869004

[13] Semova I, Carten JD, Stombaugh J, Mackey LC, Knight R, Farber SA, Rawls JF. 2012. Microbiota regulate intestinal absorption and metabolism of fatty acids in the zebrafish. Cell Host Microbe 12:277–288. doi:10.1016/j.chom.2012.08.003 22980325 PMC3517662

[14] Murdoch CC, Rawls JF. 2019. Commensal microbiota regulate vertebrate innate immunity-insights from the zebrafish. Front Immunol 10:2100. doi:10.3389/fimmu.2019.02100 31555292 PMC6742977

[15] Kanther M, Tomkovich S, Xiaolun S, Grosser MR, Koo J, Flynn EJ III, Jobin C, Rawls JF. 2014. Commensal microbiota stimulate systemic neutrophil migration through induction of serum amyloid A. Cell Microbiol 16:1053–1067. doi:10.1111/cmi.12257 24373309 PMC4364439

[16] Rolig AS, Parthasarathy R, Burns AR, Bohannan BJM, Guillemin K. 2015. Individual members of the microbiota disproportionately modulate host innate immune responses. Cell Host Microbe 18:613–620. doi:10.1016/j.chom.2015.10.009 26567512 PMC4701053

[17] Hill J. H., Franzosa EA, Huttenhower C, Guillemin K. 2016. A conserved bacterial protein induces pancreatic beta cell expansion during zebrafish development. Elife 5:e20145. doi:10.7554/eLife.20145 27960075 PMC5154760

[18] Hill JH, Massaquoi MS, Sweeney EG, Wall ES, Jahl P, Bell R, Kallio K, Derrick D, Murtaugh LC, Parthasarathy R, Remington SJ, Round JL, Guillemin K. 2022. BefA, a microbiota-secreted membrane disrupter, disseminates to the pancreas and increases β cell mass. Cell Metab 34:1779–1791. doi:10.1016/j.cmet.2022.09.001 36240759 PMC9633563

[19] Bruckner JJ, Stednitz SJ, Grice MZ, Zaidan D, Massaquoi MS, Larsch J, Tallafuss A, Guillemin K, Washbourne P, Eisen JS. 2022. The microbiota promotes social behavior by modulating microglial remodeling of forebrain neurons. PLoS Biol 20:e3001838. doi:10.1371/journal.pbio.3001838 36318534 PMC9624426

[20] Taormina MJ, Jemielita M, Stephens WZ, Burns AR, Troll JV, Parthasarathy R, Guillemin K. 2012. Investigating bacterial-animal symbioses with light sheet microscopy. Biol Bull 223:7–20. doi:10.1086/BBLv223n1p7 22983029 PMC3952068

[21] Wiles TJ, Wall ES, Schlomann BH, Hay EA, Parthasarathy R, Guillemin K. 2018. Modernized tools for streamlined genetic manipulation and comparative study of wild and diverse proteobacterial lineages. mBio 9:e01877-18. doi:10.1128/mBio.01877-18 30301859 PMC6178617

[22] Schlomann BH, Wiles TJ, Wall ES, Guillemin K, Parthasarathy R. 2018. Bacterial cohesion predicts spatial distribution in the larval zebrafish intestine. Biophys J 115:2271–2277. doi:10.1016/j.bpj.2018.10.017 30448038 PMC6289661

[23] Sundarraman D, Smith TJ, Kast JVZ, Guillemin K, Parthasarathy R. 2022. Disaggregation as an interaction mechanism among intestinal bacteria. Biophys J 121:3458–3473. doi:10.1016/j.bpj.2022.08.010 35982615 PMC9515126

[24] Schlomann BH, Parthasarathy R. 2021. Gut bacterial aggregates as living gels. Elife 10:e71105. doi:10.7554/eLife.71105 34490846 PMC8514234

[25] Wiles TJ, Schlomann BH, Wall ES, Betancourt R, Parthasarathy R, Guillemin K. 2020. Swimming motility of a gut bacterial symbiont promotes resistance to intestinal expulsion and enhances inflammation. PLoS Biol 18:e3000661. doi:10.1371/journal.pbio.3000661 32196484 PMC7112236

[26] Schlomann BH, Wiles TJ, Wall ES, Guillemin K, Parthasarathy R. 2019. Sublethal antibiotics collapse gut bacterial populations by enhancing aggregation and expulsion. Proc Natl Acad Sci USA 116:21392–21400. doi:10.1073/pnas.1907567116 31591228 PMC6815146

[27] Sundarraman D, Hay EA, Martins DM, Shields DS, Pettinari NL, Parthasarathy R. 2020. Higher-order interactions dampen pairwise competition in the zebrafish gut microbiome. mBio 11:e01667-20. doi:10.1128/mBio.01667-20 33051365 PMC7554667

[28] Stewart CJ, Ajami NJ, O’Brien JL, Hutchinson DS, Smith DP, Wong MC, Ross MC, Lloyd RE, Doddapaneni H, Metcalf GA, et al.. 2018. Temporal development of the gut microbiome in early childhood from the TEDDY study. Nature 562:583–588. doi:10.1038/s41586-018-0617-x 30356187 PMC6415775

[29] Faith JJ, Guruge JL, Charbonneau M, Subramanian S, Seedorf H, Goodman AL, Clemente JC, Knight R, Heath AC, Leibel RL, Rosenbaum M, Gordon JI. 2013. The long-term stability of the human gut microbiota. Science 341:1237439. doi:10.1126/science.1237439 23828941 PMC3791589

[30] Mehta RS, Abu-Ali GS, Drew DA, Lloyd-Price J, Subramanian A, Lochhead P, Joshi AD, Ivey KL, Khalili H, Brown GT, DuLong C, Song M, Nguyen LH, Mallick H, Rimm EB, Izard J, Huttenhower C, Chan AT. 2018. Stability of the human faecal microbiome in a cohort of adult men. Nat Microbiol 3:347–355. doi:10.1038/s41564-017-0096-0 29335554 PMC6016839

[31] Lloyd-Price J, Mahurkar A, Rahnavard G, Crabtree J, Orvis J, Hall AB, Brady A, Creasy HH, McCracken C, Giglio MG, McDonald D, Franzosa EA, Knight R, White O, Huttenhower C. 2017. Strains, functions and dynamics in the expanded human microbiome project. Nature 550:61–66. doi:10.1038/nature23889 28953883 PMC5831082

[32] Rajilić‐Stojanović M, Heilig HGHJ, Tims S, Zoetendal EG, de Vos WM. 2013. Long‐term monitoring of the human intestinal microbiota composition. Environ Microbiol 15:1146–1159. doi:10.1111/1462-2920.12023 23286720

[33] Jakobsson HE, Jernberg C, Andersson AF, Sjölund-Karlsson M, Jansson JK, Engstrand L. 2010. Short-term antibiotic treatment has differing long-term impacts on the human throat and gut microbiome. PLoS One 5:e9836. doi:10.1371/journal.pone.0009836 20352091 PMC2844414

[34] David LA, Materna AC, Friedman J, Campos-Baptista MI, Blackburn MC, Perrotta A, Erdman SE, Alm EJ. 2014. Host lifestyle affects human microbiota on daily timescales. Genome Biol 15:R89. doi:10.1186/gb-2014-15-7-r89 25146375 PMC4405912

[35] Dethlefsen L, Relman DA. 2011. Incomplete recovery and individualized responses of the human distal gut microbiota to repeated antibiotic perturbation. Proc Natl Acad Sci USA 108:4554–4561. doi:10.1073/pnas.1000087107 20847294 PMC3063582

[36] Schlomann BH, Parthasarathy R. 2019. Timescales of gut microbiome dynamics. Curr Opin Microbiol 50:56–63. doi:10.1016/j.mib.2019.09.011 31689582 PMC6899164

[37] Forberg T, Milligan-Myhre K. 2017. Gnotobiotic fish as models to study host–microbe interactions, p 369–383. In Gnotobiotics. Elsevier.

[38] McElligott MB, O’malley DM. 2005. Prey tracking by larval zebrafish: axial kinematics and visual control. Brain Behav Evol 66:177–196. doi:10.1159/000087158 16088102

[39] Mearns DS, Donovan JC, Fernandes AM, Semmelhack JL, Baier H. 2020. Deconstructing hunting behavior reveals a tightly coupled stimulus-response loop. Curr Biol 30:54–69. doi:10.1016/j.cub.2019.11.022 31866365

[40] Provasoli L, Shiraishi K. 1959. Axenic cultivation of the brine shrimp artemia salina. Biol Bull 117:347–355. doi:10.2307/1538914

[41] de la Torre Canny SG, Mueller O, Craciunescu CV, Blumberg B, Rawls JF. 2021. Tributyltin exposure leads to increased adiposity and reduced abundance of leptogenic bacteria in the zebrafish intestine. bioRxiv. doi:10.1101/2021.07.09.451869

[42] Rendueles O, Ferrières L, Frétaud M, Bégaud E, Herbomel P, Levraud J-P, Ghigo J-M. 2012. A new zebrafish model of oro-intestinal pathogen colonization reveals a key role for adhesion in protection by probiotic bacteria. PLoS Pathog 8:e1002815. doi:10.1371/journal.ppat.1002815 22911651 PMC3406073

[43] Lenhoff HM. 1983. Hatching brine shrimp larvae axenically and/or in a range of quantities, p 39–46. In Hydra: research methods. Springer.

[44] Jia P-P, Yang Y-F, Li W-G, Duan J-J, Wang Y, Pei D-S. 2023. Breaking the mold: the first report on germ-free adult marine medaka (Oryzias melastigma) models. bioRxiv. doi:10.1101/2023.04.10.536225

[45] Limbu SM, Chen L-Q, Zhang M-L, Du Z-Y. 2021. A global analysis on the systemic effects of antibiotics in cultured fish and their potential human health risk: a review. Rev Aquacult 13:1015–1059. doi:10.1111/raq.12511

[46] Dhert P, Rombaut G, Suantika G, Sorgeloos P. 2001. Advancement of rotifer culture and manipulation techniques in Europe. Aquacult 200:129–146. doi:10.1016/S0044-8486(01)00697-4

[47] Harper C, Lawrence C. 2016. The laboratory zebrafish. CRC Press.

[48] Dougherty EC, Solberg B, Harris LG. 1960. Synxenic and attempted axenic cultivation of rotifers. Science 132:416–417. doi:10.1126/science.132.3424.416 17756970

[49] Tinh NTN, Dierckens K, Sorgeloos P, Bossier P. 2006. Gnotobiotically grown rotifer Brachionus plicatilis sensu strictu as a tool for evaluation of microbial functions and nutritional value of different food types. Aquacult 253:421–432. doi:10.1016/j.aquaculture.2005.09.006

[50] Chang CT, Benedict S, Whipps CM. 2019. Transmission of Mycobacterium chelonae and Mycobacterium marinum in laboratory zebrafish through live feeds. J Fish Dis 42:1425–1431. doi:10.1111/jfd.13071 31418901 PMC6744340

[51] Munro PD, Henderson RJ, Barbour A, Birkbeck TH. 1999. Partial decontamination of rotifers with ultraviolet radiation: the effect of changes in the bacterial load and flora of rotifers on mortalities in start-feeding larval turbot. Aquacult 170:229–244. doi:10.1016/S0044-8486(98)00419-0

[52] Cho I, Yamanishi S, Cox L, Methé BA, Zavadil J, Li K, Gao Z, Mahana D, Raju K, Teitler I, Li H, Alekseyenko AV, Blaser MJ. 2012. Antibiotics in early life alter the murine colonic microbiome and adiposity. Nature 488:621–626. doi:10.1038/nature11400 22914093 PMC3553221

[53] Rolig AS, Sweeney EG, Kaye LE, DeSantis MD, Perkins A, Banse AV, Hamilton MK, Guillemin K. 2018. A bacterial immunomodulatory protein with lipocalin-like domains facilitates host-bacteria mutualism in larval zebrafish. Elife 7:e37172. doi:10.7554/eLife.37172 30398151 PMC6219842

[54] Lam SH, Chua HL, Gong Z, Lam TJ, Sin YM. 2004. Development and maturation of the immune system in zebrafish, Danio rerio: a gene expression profiling, in situ hybridization and immunological study. Devel Comp Immun 28:9–28. doi:10.1016/S0145-305X(03)00103-4 12962979

[55] Ruff WE, Greiling TM, Kriegel MA. 2020. Host-microbiota interactions in immune-mediated diseases. Nat Rev Microbiol 18:521–538. doi:10.1038/s41579-020-0367-2 32457482

[56] Westerfield M. 2007. A guide for the laboratory use of zebrafish (*Danio rerio*). In The zebrafish book. University of Oregon Press.

[57] Parthasarathy R. 2012. Rapid, accurate particle tracking by calculation of radial symmetry centers. Nat Methods 9:724–726. doi:10.1038/nmeth.2071 22688415

[58] Keller PJ, Schmidt AD, Wittbrodt J, Stelzer EHK. 2008. Reconstruction of zebrafish early embryonic development by scanned light sheet microscopy. Science 322:1065–1069. doi:10.1126/science.1162493 18845710

[59] Jemielita M, Taormina MJ, Burns AR, Hampton JS, Rolig AS, Guillemin K, Parthasarathy R. 2014. Spatial and temporal features of the growth of a bacterial species colonizing the zebrafish gut. mBio 5:e01751-14. doi:10.1128/mBio.01751-14 25516613 PMC4271548

[60] Wiles TJ, Jemielita M, Baker RP, Schlomann BH, Logan SL, Ganz J, Melancon E, Eisen JS, Guillemin K, Parthasarathy R. 2016. Host gut motility promotes competitive exclusion within a model intestinal microbiota. PLoS Biol 14:e1002517. doi:10.1371/journal.pbio.1002517 27458727 PMC4961409

[61] Logan SL, Thomas J, Yan J, Baker RP, Shields DS, Xavier JB, Hammer BK, Parthasarathy R. 2018. The Vibrio cholerae type VI secretion system can modulate host intestinal mechanics to displace gut bacterial symbionts. Proc Natl Acad Sci U S A 115:E3779–E3787. doi:10.1073/pnas.1720133115 29610339 PMC5910850

