# Supplementary material for: UV-irradiated rotifers for the maintenance of gnotobiotic zebrafish larvae
Source: mSphere. 2025 Feb 24;10(3):e00698-24. doi: 10.1128/msphere.00698-24 (PMC11934316; doi:10.1128/msphere.00698-24)
Supplement: Supplemental Figures and Movie Captions — Figures S1 and S2 and captions for Movies S1 and S2. [file msphere.00698-24-s0001.pdf]

Supplemental Material

UV-irradiated rotifers for the maintenance of gnotobiotic zebrafish larvae

Susana Márquez Rosales[1], Peter I. Bouchard[1], Emily M. Olmstead[1], Raghuveer Parthasarathy[1]*

[1]Department of Physics, Institute of Molecular Biology, and Materials Science Institute, University of Oregon, Eugene, Oregon, 97403, USA
*Address correspondence to Raghuveer Parthasarathy, raghu@uoregon.edu.

Contents

- Supplemental Movie Captions.
- Supplemental Figures and Captions.

Supplemental Movie Caption

Supplemental Movie 1. Representative movie of rotifers without UV irradiation. Rotifer motility is evident, as are a large number of dead rotifers. Brightfield, 10 fps.

Supplemental Movie 2. Representative movie of rotifers following four cycles of UV irradiation, as described in the text. Rotifer motility at reduced speed compared to control rotifers is evident, as are a large number of dead rotifers. Brightfield, 10 fps.

Supplemental Figures

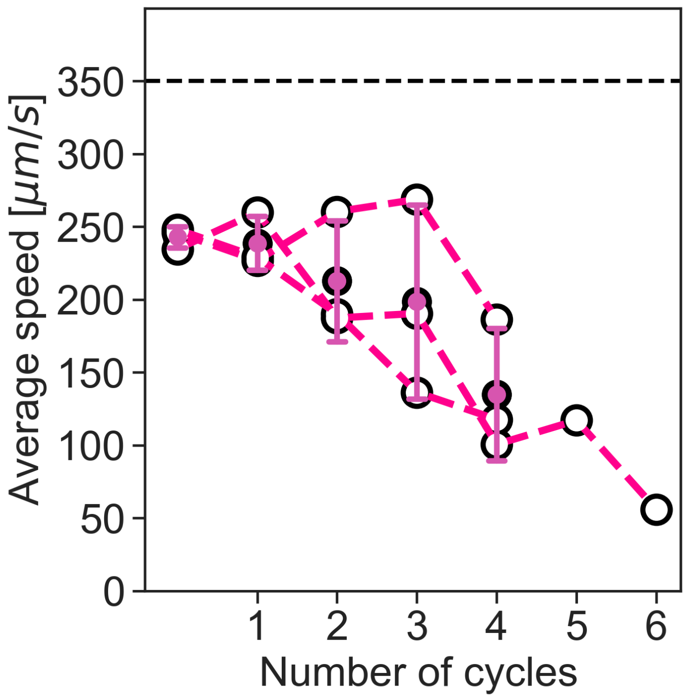

Supplemental Figure S1. Average speed of motile rotifers as a function of the number of UV exposure cycles, each 30 minutes on / 30 minutes off. Dotted line at 350µm/s represents the average speed of rotifers before UV irradiation. Open symbols represent different replicates, and solid symbols and error bars indicate the mean and standard deviation.

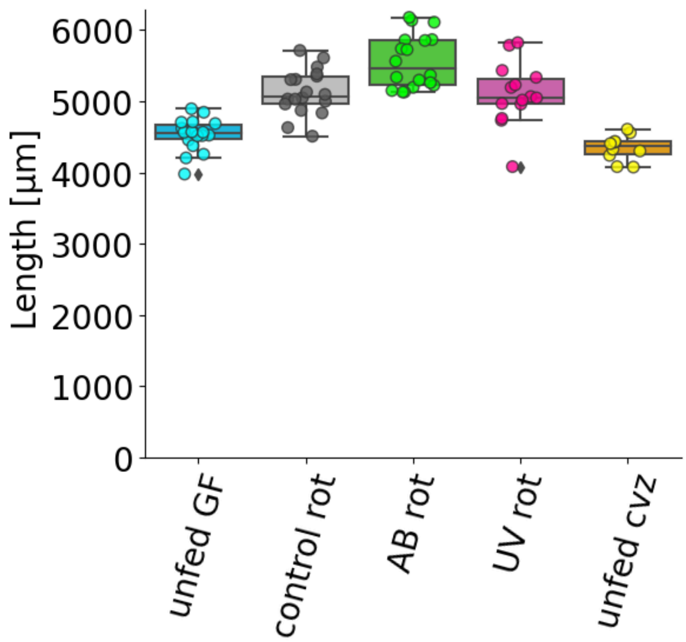

Supplemental Figure S2. Fish length on 7 or 8 dpf after 2 or 3 days of feeding with rotifers under different treatments, or unfed at the same days. Each symbol indicates a measurement from an individual fish; boxes indicate the median (line within the box), first and third quartiles (top and bottom of the box), and 95\% confidence intervals.
